# Syndecan-1, an indicator of endothelial glycocalyx degradation, predicts outcome of patients admitted to an ICU with COVID-19

**DOI:** 10.1186/s10020-021-00412-1

**Published:** 2021-12-03

**Authors:** Dong Zhang, Liubing Li, Yu Chen, Jie Ma, Yanli Yang, Surita Aodeng, Qiuju Cui, Kedi Wen, Meng Xiao, Jing Xie, Yingchun Xu, Yongzhe Li

**Affiliations:** 1 Department of Clinical Laboratory, Peking Union Medical College Hospital, Peking Union Medical College and Chinese Academy of Medical Sciences, Beijing, 100730 People’s Republic of China; 2 Division of Nephrology, Department of Internal Medicine, Peking Union Medical College Hospital, Peking Union Medical College and Chinese Academy of Medical Sciences, Beijing, 100730 People’s Republic of China; 3 Department of Respiratory and Critical Care Medicine, Peking Union Medical College Hospital, Peking Union Medical College and Chinese Academy of Medical Sciences, Beijing, 100730 People’s Republic of China; 4 Department of Otolaryngology, Peking Union Medical College Hospital, Peking Union Medical College and Chinese Academy of Medical Sciences, Beijing, 100730 People’s Republic of China; 5 Operating Room, Peking Union Medical College Hospital, Peking Union Medical College and Chinese Academy of Medical Sciences, Beijing, 100730 People’s Republic of China; 6 Department of Infectious Diseases, Peking Union Medical College Hospital, Peking Union Medical College and Chinese Academy of Medical Sciences, Beijing, 100730 People’s Republic of China

**Keywords:** Syndecan-1, Endothelial glycocalyx, Degradation, Outcome, COVID-19

## Abstract

**Background:**

We investigated the feasibility of two biomarkers of endothelial damage (Syndecan-1 and thrombomodulin) in coronavirus disease 2019 (COVID-19), and their association with inflammation, coagulopathy, and mortality.

**Methods:**

The records of 49 COVID-19 patients who were admitted to an intensive care unit (ICU) in Wuhan, China between February and April 2020 were examined. Demographic, clinical, and laboratory data, and outcomes were compared between survivors and non-survivors COVID-19 patients, and between patients with high and low serum Syndecan-1 levels. The dynamics of serum Syndecan-1 levels were also analyzed.

**Results:**

The levels of Syndecan-1 were significantly higher in non-survivor group compared with survivor group (median 1031.4 versus 504.0 ng/mL, P = 0.002), and the levels of thrombomodulin were not significantly different between these two groups (median 4534.0 versus 3780.0 ng/mL, P = 0.070). Kaplan–Meier survival analysis showed that the group with high Syndecan-1 levels had worse overall survival (log-rank test: P = 0.023). Patients with high Syndecan-1 levels also had significantly higher levels of thrombomodulin, interleukin-6, and tumor necrosis factor-α. Data on the dynamics of Syndecan-1 levels indicated much greater variations in non-survivors than survivors.

**Conclusions:**

COVID-19 patients with high levels of Syndecan-1 develop more serious endothelial damage and inflammatory reactions, and have increased mortality. Syndecan-1 has potential for use as a marker for progression or severity of COVID-19. Protecting the glycocalyx from destruction is a potential treatment for COVID-19.

**Supplementary Information:**

The online version contains supplementary material available at 10.1186/s10020-021-00412-1.

## Introduction

Coronavirus disease 2019 (COVID-19), which is caused by the severe acute respiratory syndrome coronavirus 2 (SARS-CoV-2), has become a critical problem in many countries. Similar to SARS-CoV-1, research indicates that SARS-CoV-2 enters human cells by binding to angiotensin converting enzyme 2 (ACE2) (Zhou et al. 2020; Wan et al. 2020), a receptor present on the endothelial cells of many tissues, such as the lungs, heart, and kidneys (Zhang et al. 2020). ACE2 functions as a carboxypeptidase, and it cleaves angiotensin II (Ang-II) into Ang (Zhou et al. 2020; Wan et al. 2020; Zhang et al. 2020; Tipnis et al. 2000; Han et al. 2018; Boegehold et al. 2015; Ackermann et al. 2020) and degrades Ang-I into Ang (Zhou et al. 2020; Wan et al. 2020; Zhang et al. 2020; Tipnis et al. 2000; Han et al. 2018; Boegehold et al. 2015; Ackermann et al. 2020; Su et al. 2020; Johansson et al. 2011). An elevated level of Ang-II increases the production of superoxide anion, thereby increasing oxidative stress, dysfunction, and damage of endothelial cells (Han et al. 2018; Boegehold et al. 2015). Thus, the binding of SARS-CoV-2 to ACE2 may lead to Ang-II-induced endothelial injury, and which has been observed in lung, kidney and other organs at autopsies in COVID-19 patients (Ackermann et al. 2020; Su et al. 2020).

Syndecan-1 and thrombomodulin are biomarkers of endothelial function. Syndecan-1 is a heparan sulfate proteoglycan expressed in endothelial cells and the main marker of endothelial glycocalyx degradation (Johansson et al. 2011). An elevated serum level of Syndecan-1 is associated with endothelial injury (Ito et al. 2019; Loghmani and Conway 2018). Thrombomodulin is a type I transmembrane glycoprotein that is present on the luminal surfaces of endothelial cells. The measurement of soluble thrombomodulin may represent early manifestations of endothelial dysfunction. Recent studies reported the levels of Syndecan-1 and thrombomodulin in COVID-19 patients (Karampoor 2021; Juneja et al. 2021; Suzuki et al. 2021; Bouck et al. 2021; Fraser 2020a; Goshua et al. 2020). However, there are controversies regarding the association between these biomarkers and endothelial damage status in COVID-19 patients. For example, Fraser et al. showed that intensive care unit (ICU) patients with COVID-19 had high Syndecan-1 (Fraser 2020a). Conversely, Hutchings et al. reported that Syndecan-1 levels were marginally elevated in critically ill patients with COVID-19 compared to healthy controls but overall most patients did not have markedly elevated Syndecan-1 levels (Hutchings et al. 2021).

Endothelial dysfunction is a crucial involved pathology in COVID-19 which leads to poor outcomes (Norooznezhad and Mansouri 2021). Therefore, it is important to clarify the feasibility of biomarkers of endothelial damage for the assessment of endothelial function in COVID-19. This study aimed to determine the prognostic values of endothelial damage biomarkers (Syndecan-1 and thrombomodulin) in COVID-19 patients in China, as well as their associations with inflammation, coagulopathy, and mortality.

## Methods

### Patients

Forty-nine adult patients diagnosed with COVID-19 according to the Diagnosis and Treatment Protocol for Novel Coronavirus Pneumonia (Trial Version 7) were included in this retrospective study. Adult patients who meet any of the following criteria are defined as severe cases: (1) respiratory distress (≧ 30 breaths/min); (2) oxygen saturation ≤ 93% at rest; (3) arterial partial pressure of oxygen (PaO_2_)/fraction of inspired oxygen (FiO_2_) ≦ 300 mmHg (l mmHg = 0.133 kPa). Patients who meet any of the following criteria are defined as critical severe cases: (1) respiratory failure occurs and mechanical ventilation is required; (2) shock occurs; (3) combined with the failure of other organs, and ICU monitoring and treatment is required. All data were from patients who were admitted to an ICU in the Sino-French New City Branch of Tongji Hospital (Wuhan, China) between February 2020 and April 2020. The demographic features, clinical characteristics, treatments, outcomes and laboratory information of all patients were collected. This study was approved by the Research Ethics Commission of PUMCH and the requirement for informed consent was waived by the Ethics Commission because the study was retrospective.

### Confirmation of SARS-CoV-2 infection

Nasopharyngeal swab samples were collected from each participant. SARS-CoV-2 was detected using a commercial reverse-transcriptase polymerase-chain-reaction (RT-PCR) kit (BGI Biotechnology Co, Ltd., Wuhan, China). The detection of anti-SARS-CoV-2 antibodies (IgM and IgG) was performed using a commercial immunochromatographic assay (Beijing Hotgen Biotech Co., Ltd, Beijing, China).

### Collection of blood samples

Blood samples were collected from a COVID-19 cohort where recruitment took place on the day of admission to ICU. Subsequent samples were obtained from patients in the morning. Blood samples were collected in procoagulant tubes containing separating gel before being spun in a centrifuge at 4500 rpm for 15 min. Serum was aliquoted and frozen at − 80 °C. All samples remained frozen until use and freeze/thaw cycles were minimized.

### Determination of biomarkers of endothelial damage/activation and cytokines

Serum samples from 49 patients with COVID-19 were assayed for the presence of Syndecan-1 (Abcam, Cambridge, UK) and thrombomodulin (Beijing 4A Biotech Co., Ltd, Beijing, China) using enzyme-linked immunosorbent assay (ELISA) kits according to each manufacturer’s instruction. The measurement of Syndecan-1 and thrombomodulin was repeated twice for each sample, and the mean value was taken as the finally determined value. The measurement of cytokines was performed using the BD™ Cytometric Bead Array Human Th1/Th2 Cytokine kit II (BD Biosciences, Franklin Lakes, NJ, USA) as previously described (Sciammarella et al. 2020). Each sample was processed in triplicate and the data were expressed as mean ± SD.

### Statistics

Data were plotted using GraphPad Prism 5 (San Diego, CA, USA). Receiver operating characteristic (ROC) curves were plotted and the maximal cut-off was determined by calculating the Youden index. Kaplan–Meier analysis was used to assess survival status. Statistical analysis was performed using SPSS version 20.0 (Chicago, USA). For comparisons of continuous variables, Student’ s *t*-test or the Mann-U-test were used, as appropriate. For comparisons of categorical variables, the Chi-square test was used. Bivariate associations between variables of interest were assessed by Sperman rank correlations. A *P* value less than 0.05 was considered significant.

## Results

### Baseline characteristics of COVID-19 patients

We retrospectively examined the records of 49 patients with COVID-19 who were admitted to our ICU within a 3-month period (Table 1). The median age was 65.0 years (Q1–Q3: 56.5–72.0) and 65.3% were male. The most common comorbidities were hypertension (49.0%), cardiovascular disease (24.5%), and diabetes (16.3%). The most common symptoms at enrollment were fever (85.7%), cough (69.4%), dyspnea (61.2%), fatigue (55.1%), and sputum production (55.1%). Among all 49 patients, 11 (22.4%) had severe disease and 38 (77.6%) had critically severe disease. Fourteen patients (28.6%) survived and were discharged from the ICU and the other 35 patients (71.4%) died.

**Table 1:** Baseline characteristics of survivor and non-survivor COVID-19 patients at ICU admission

	Total (N = 49)	COVID-19 patients		P
		Non-survivors (N = 35)	Survivors (N = 14)	
Age, years	65.0 (56.5–72.0) (49)	65.0 (59.0–73.0) (35)	56.5 (53.8–70.8) (14)	**0.020**
Gender				
Male/female	65.3% (32/49)/34.7% (17/49)	74.3% (26/35)/25.7% (9/35)	42.9% (6/14)/57.1% (8/14)	0.079
Comorbidities/condition				
Smoking	10.2% (5/49)	11.4% (4/35)	7.1% (1/14)	1.000
Hypertension	49.0% (24/49)	48.6% (17/35)	50.0% (7/14)	0.928
Diabetes	16.3% (8/49)	11.4% (4/35)	28.6% (4/14)	0.299
Cardiovascular disease	24.5% (12/49)	25.7% (9/35)	21.4% (3/14)	1.000
Cerebrovascular disease	14.3% (7/49)	11.4% (4/35)	21.4% (3/14)	0.651
Chronic lung disease	4.1% (2/49)	5.7% (2/35)	0.0% (0/14)	1.000
Chronic kidney disease	6.1% (3/49)	2.9% (1/35)	14.3% (2/14)	0.193
Chronic liver disease	2.0% (1/49)	2.9% (1/35)	0.0% (0/14)	1.000
Anemia	4.1% (2/49)	0.0% (0/35)	14.3% (2/14)	0.077
Malignance	4.1% (2/49)	5.7% (2/35)	0.0% (0/14)	1.000
Autoimmune diseases	2.0% (1/49)	0.0% (0/35)	7.1% (1/14)	0.286
Symptoms and signs				
Fever	85.7% (42/49)	85.7% (30/35)	85.7% (12/14)	1.000
Fatigue	55.1% (27/49)	57.1% (20/35)	50.0% (7/14)	0.650
Dyspnea	61.2% (30/49)	68.6% (24/35)	42.9% (6/14)	0.095
Cough	69.4% (34/49)	68.6% (24/35)	71.4% (10/14)	1.000
Sputum	55.1% (27/49)	54.3% (19/35)	57.1% (8/14)	0.856
Pharyngeal pain	14.3% (7/49)	17.1% (6/35)	7.1% (1/14)	0.651
Abdominal pain	24.5% (12/49)	28.6% (10/35)	14.3% (2/14)	0.495
Diarrhea	30.6% (15/49)	37.1% (13/35)	14.3% (2/14)	0.220
Headache	20.4% (10/49)	25.7% (9/35)	7.1% (1/14)	0.287
Dizziness	8.2% (4/49)	11.4% (4/35)	0.0% (0/14)	0.458
Nausea	28.6% (14/49)	34.3% (12/35)	14.3% (2/14)	0.294
Vomiting	24.5% (12/49)	28.6% (10/35)	14.3% (2/14)	0.495
Anorexia	20.4% (10/49)	25.7% (9/35)	7.1% (1/14)	0.287
Myalgia	20.4% (10/49)	22.9% (8/35)	14.3% (2/14)	0.779
Disease severity status				
Severe/Critically severe	22.4% (11/49)/77.6% (38/49)	0.0% (0/35)/100.0% (35/35)	78.8% (11/14)/21.4% (3/14)	** < 0.001**
Treatment				
MV	66.7% (32/48)	85.3% (29/34)	21.4% (3/14)	** < 0.001**
Invasive MV	90.6% (29/32)	89.7% (26/29)	100% (3/3)	1.000
PEEP (cm H_2_O)	12.0 (10.0–14.0) (29)	12.0 (10.0–14.0) (26)	14.0 (NA) (3)	0.948
PaCO_2_ (mmHg)	49.0 (42.0–58.8) (28)	49.0 (42.0–58.5) (21)	49.0 (47.0–64.0) (7)	0.876
Oxygenation index (mmHg)	135.5 (84.2–276.5) (23)	117.9 (79.4–197.1) (19)	311.7 (286.5–356.8) (4)	**0.010**
Lactate (mmol/L)	21.0 (7.0–34.0) (16)	23.0 (15.5–34.0) (12)	7.0 (5.0–33.0) (4)	0.350

Bold values indicate statistical significance

Data are presented as median (Q1–Q3) (N) or % (n/N)

COVID-19, coronavirus disease 2019; MV, mechanical ventilation; PEEP, positive end-expiratory pressure; PaCO_2_, partial pressure of carbon dioxide

### Comparison of baseline characteristics between survivors and non-survivors COVID-19 patients

Patients in non-survivor group were older (median 65.0 versus 56.5 years, P = 0.020, Table 1) and more severely ill on ICU admission (critically severe: 100% versus 21.4%, P < 0.001, Table 1) than those in survivor group. Mechanical ventilation was more required (85.3% versus 21.4%, P < 0.001, Table 1) and oxygenation index was lower (median 117.9 versus 311.7 mmHg, P = 0.010, Table 1) in non-survivors compared with survivor group.

### Comparison of laboratory characteristics between survivors and non-survivors COVID-19 patients

**Fig. 1:**
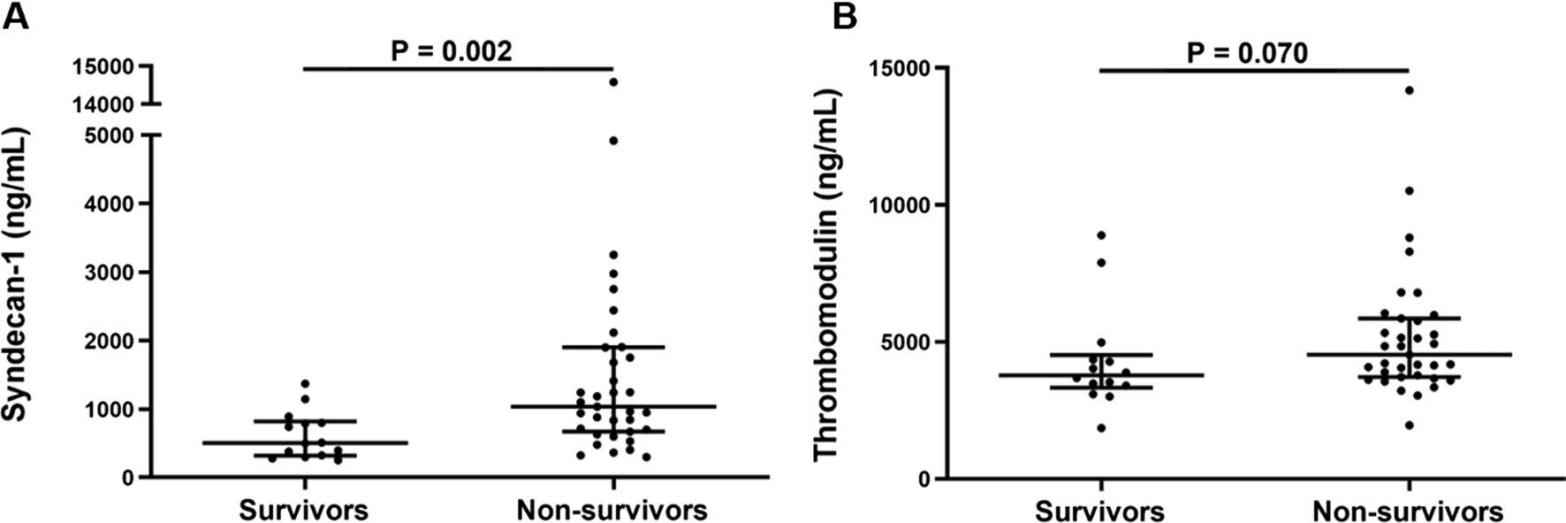
Levels of syndecan-1 and thrombomodulin in survivors and non-survivors COVID-19 patients.** A** The levels of syndecan-1 were significantly higher in non-survivor group compared with survivor group. **B** The levels of thrombomodulin were not significantly different between non-survivor group and survivor group

The levels of Syndecan-1 were significantly higher in non-survivor group compared with survivor group (median 1031.4 *versus* 504.0 ng/mL, P = 0.002, Fig. 1A), and the levels of thrombomodulin were not significantly different between these two groups (median 4534.0 versus 3780.0 ng/mL, P = 0.070, Fig. 1B). Compared to the survivor group, non-survivors had higher interleukin (IL)-6, IL-8, neutrophil count, high-sensitivity C-reactive protein (hsCRP), myoglobin, prothrombin time, international normalized ratio (INR), D-dimer, fibrinogen degradation products and procalcitonin (Table 2), and had lower lymphocyte count, platelet count, total cholesterol and prothrombin activity (Table 2). The normal reference ranges of laboratory parameters were listed in Additional file 1: Table S1.

**Table 2:** Laboratory findings of survivor and non-survivor COVID-19 patients at ICU admission

	Total (N = 49)	COVID-19 patients		P
		Non-survivors (N = 35)	Survivors (N = 14)	
Tissue and endothelial damage/activation				
Syndecan-1 (ng/mL)	880.3 (504.0–1387.9) (49)	1031.4 (669.7–1901.7) (35)	504.0 (316.0–819.8) (14)	**0.002**
Thrombomodulin (ng/mL)	4175.0 (3604.3–5550.9) (49)	4534.0 (3722.5–5858.4) (35)	3780.0 (3326.9–4514.3) (14)	0.070
Inflammation				
IL-1β (pg/mL)↑	15.8% (3/19)	15.4% (2/13)	16.7% (1/6)	1.000
IL-2R (U/mL)	1065.5 (493.8–1536) (20)	1284.0 (670.0–1981.0) (13)	1052.0 (359.0–1111.0) (7)	0.183
IL-6 (pg/mL)	97.7 (12.6–179.0) (25)	139.3 (66.2–294.8) (16)	12.2 (6.1–33.8) (9)	** < 0.001**
IL-8 (pg/mL)	51.7 (12.1–93.3) (19)	79.3 (42.5–163.0) (13)	15.7 (9.4–23.4) (6)	**0.005**
IL-10 (pg/mL)	9.2 (2.5–20.7) (19)	16.5 (2.5–31.6) (13)	4.3 (2.5–14.8) (6)	0.179
TNFα (pg/mL)	9.5 (6.2–24.0) (17)	14.5 (2.1–30.5) (11)	7.6 (6.2–9.5) (6)	0.494
Hematologic				
White blood cell count (× 10^9^/L)	10.9 (7.6–17.7) (46)	12.2 (7.9–18.7) (34)	9.5 (6.7–11.1) (12)	0.133
Neutrophil count (× 10^9^/L)	8.8 (6.1–16.5) (45)	11.3 (6.8–17.8) (34)	6.7 (5.1–8.7) (11)	**0.039**
Lymphocyte count (× 10^9^/L)	0.5 (0.3–0.8) (47)	0.5 (0.3–0.7) (34)	1.0 (0.5–1.7) (13)	**0.012**
Red blood cell count (× 10^12^/L)↓	0.0% (0/46)	0.0% (0/34)	0.0% (0/12)	NA
Hemoglobin (g/L)↓	0.0% (0/47)	0.0% (0/34)	0.0% (0/13)	NA
Hematocrit (%)↓	0.0% (0/46)	0.0% (0/34)	0.0% (0/12)	NA
Platelet count (× 10^9^/L)	112.5 (55.5–213.5) (46)	75.5 (43.3–141.5) (34)	238.5 (148.8–349.8) (12)	** < 0.001**
Biochemical				
Glucose (mmol/L)	9.9 (7.2–12.6) (45)	10.9 (8.4–14.5) (33)	7.8 (6.2–10.3) (12)	0.057
Total cholesterol (mmol/L)	3.1 (2.4–3.5) (46)	2.8 (2.3–3.5) (34)	3.3 (3.1–5.0) (12)	**0.027**
HsCRP (mg/L)	79.4 (49.0–186.9) (35)	126.9 (61.9–205.1) (25)	31.6 (7.2–76.6) (10)	** < 0.001**
High-sensitive cardiac troponin I (pg/mL)↑	68.9% (31/45)	75.0% (24/32)	53.8% (7/13)	0.301
Myoglobin (ng/mL)↑	48.9% (22/45)	59.4% (19/32)	23.1% (3/13)	**0.027**
AST (U/L)↑	40.0% (18/45)	41.2% (14/34)	36.4% (4/11)	1.000
LDH (U/L)↑	93.6% (44/47)	97.1% (33/34)	84.6% (11/13)	0.181
CK (U/L)↑	38.1% (8/21)	44.4% (8/18)	0.0% (0/3)	0.409
CK-MB (ng/mL)↑	28.9% (13/45)	37.5% (12/32)	7.7% (1/13)	0.102
NT-proBNP (pg/mL)↑	84.1% (37/44)	87.1% (27/31)	76.9% (10/13)	0.696
Ferritin (μg/L)↑	90.9% (10/11)	100.0% (6/6)	80.0% (4/5)	0.455
Coagulation				
Prothrombin time (second)	15.9 (14.9–18.7) (45)	17.3 (15.7–21.0) (33)	14.2 (13.6–15.7) (12)	** < 0.001**
Prothrombin activity (%)	68.5 (51.8–78.0) (44)	59.0 (44.0–71.0) (33)	88.0 (71.0–95.0) (11)	** < 0.001**
INR (ratio)	1.3 (1.2–1.6) (44)	1.4 (1.2–1.8) (33)	1.1 (1.0–1.2) (11)	** < 0.001**
Fibrinogen (g/L)	3.9 (3.2–5.3) (45)	3.6 (2.8–5.5) (33)	4.5 (3.6–5.3) (12)	0.495
APTT (second)	42.6 (38.0–51.9) (45)	42.6 (38.5–54.4) (33)	41.6 (36.5–45.8) (12)	0.303
Thrombin time (second)	15.5 (14.7–16.8) (45)	15.5 (14.8–17.5) (33)	15.4 (14.6–16.0) (12)	0.470
D-dimer (μg/mL FEU)	7.1 (2.5–18.6) (44)	13.5 (3.9–21.0) (33)	2.4 (1.2–3.7) (11)	**0.002**
Fibrinogen degradation products (μg/mL)	54.7 (13.7–110.9) (19)	56.7 (17.0–150.0) (15)	8.0 (4.0–44.3) (4)	**0.027**
Antithrombin (%)	82.0 (69.0–90.0) (23)	82.0 (66.5–87.0) (17)	86.5 (70.8–96.5) (6)	0.392
Other				
Procalcitonin (ng/mL)	0.34 (0.10–1.32) (22)	0.52 (0.28–3.12) (13)	0.12 (0.07–0.30) (9)	**0.006**
ESR (mm/H)↑	88.9% (8/9)	75.0% (3/4)	100.0% (5/5)	0.444
aPLs positive	50.0% (3/6)	50.0% (2/4)	50.0% (1/2)	1.000

Bold values indicate statistical significance

Data are presented as median (Q1–Q3) (N) or % (n/N)

COVID-19, coronavirus disease 2019; IL, interleukin; TNF-α, tumor necrosis factor-alpha; hsCRP, high-sensitivity C-reactive protein; AST, aspartate transaminase; LDH, lactate dehydrogenase; CK, creatine kinase; CK-MB, creatine kinase-myoglobin band; NT-proBNP, N-terminal pro-brain natriuretic peptide; INR, international normalized ratio; APTT, activated partial thromboplastin time; ESR, erythrocyte sedimentation rate; aPLs, antiphospholipid antibodies

### Prognostic values of Syndecan-1 in COVID-19 patients

**Fig. 2:**
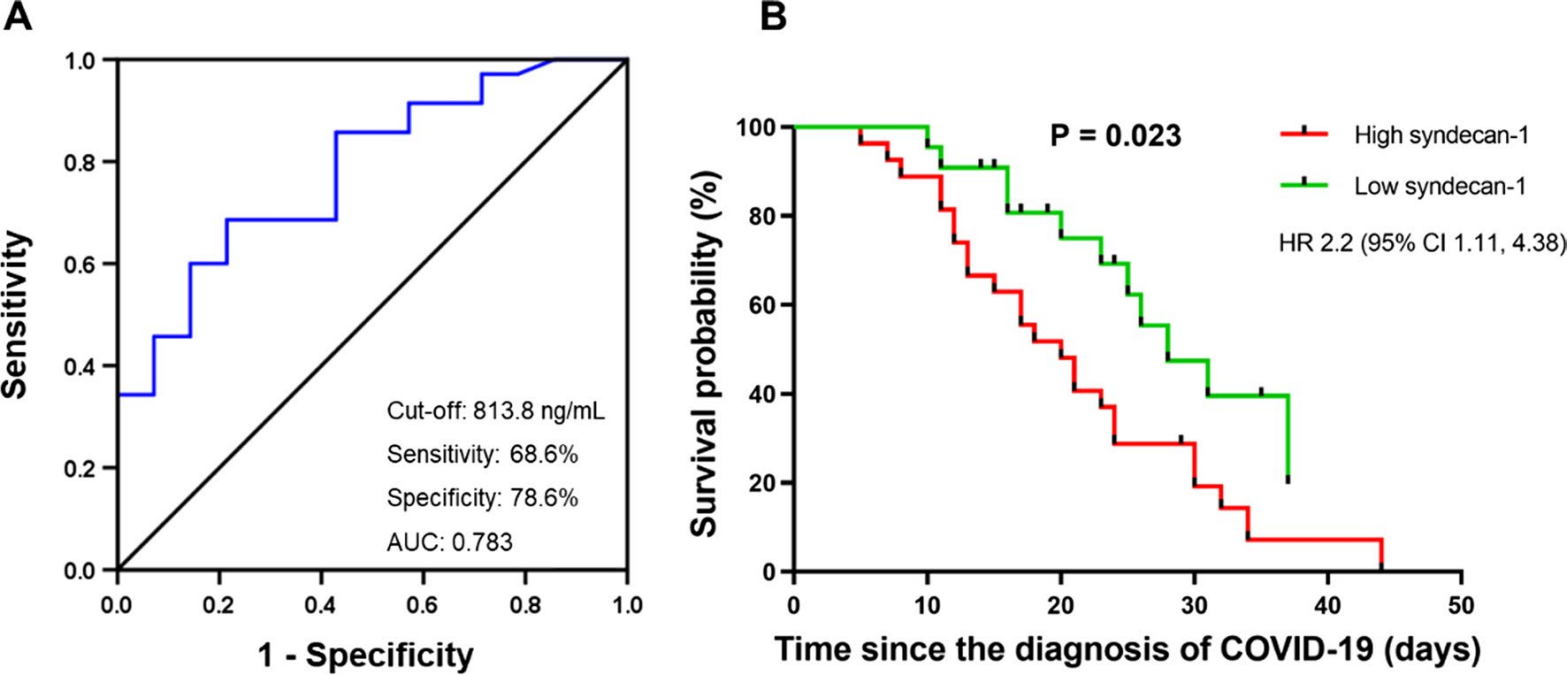
Receiver operating characteristic analysis and Kaplan–Meier survival analysis.** A** Receiver operating characteristic analysis of Syndecan-1 levels in distinguishing non-survivors from survivors. **B** Kaplan–Meier survival analysis of COVID-19 patients with high *versus* low syndecan-1 levels

The ROC analysis revealed an optimal cut-off value of Syndecan-1 (813.8 ng/mL) to distinguish non-survivors from survivors, with a sensitivity of 68.6% and specificity of 78.6% and an area under curve (AUC) of 0.783 (95% confidence interval [CI] 0.647–0.918, P = 0.002) (Fig. 2A). Furthermore, COVID-19 patients were divided into high and low Syndecan-1 groups according to the cut-off value. Kaplan–Meier analysis indicated a significantly worse overall survival in patients with high levels of Syndecan-1 (log-rank test: P = 0.023, Gehan-Breslow-Wilcoxon test: P = 0.019, hazard ratio [HR]: 2.2, 95% CI 1.11–4.38) (Fig. 2B).

### Characteristics of patients with high versus low levels of Syndecan-1

All 22 patients (100%) with low levels of Syndecan-1 and 20 of 27 patients (74.1%) with high levels of Syndecan-1 presented with fever (P = 0.030, Table 3). Further analysis of clinical data (Table 3) indicated that patients with high levels of Syndecan-1 had more requirement for mechanical ventilation and significantly poorer prognosis compared with those with low levels of Syndecan-1. Further analysis of laboratory data (Table 4) showed that patients in the high Syndecan-1 group had higher thrombomodulin, IL-6, tumor necrosis factor α (TNFα), hsCRP, myoglobin, creatinine kinase myocardial band (CK-MB), prothrombin time, INR, activated partial thromboplastin time (APTT) and procalcitonin, and had lower levels of platelet count, total cholesterol and prothrombin activity.

**Table 3:** Demographics, clinical characteristics, treatment, and outcome of COVID-19 patients stratified by Syndecan-1 level at ICU admission (cutoff: 813.8 ng/mL)

	Syndecan-1		P
	High level (N = 27)	Low level (N = 22)	
Age, years	64.0 (57.0–71.0) (27)	65.5 (56.0–73.0) (22)	0.984
Gender			
Male/female	74.1% (20/27)/25.9% (7/27)	54.5% (12/22)/45.5% (10/22)	0.153
Comorbidities/conditions			
Smoking	11.1% (3/27)	9.1% (2/22)	1.000
Hypertension	48.1% (13/27)	50.0% (11/22)	0.897
Diabetes	7.4% (2/27)	27.3% (6/22)	0.138
Cardiovascular disease	18.5% (5/27)	31.8% (7/22)	0.282
Cerebrovascular disease	14.8% (4/27)	13.6% (3/22)	1.000
Chronic lung disease	3.7% (1/27)	4.5% (1/22)	1.000
Chronic kidney disease	3.7% (1/27)	9.1% (2/22)	0.855
Chronic liver disease	0.0% (0/27)	4.5% (1/22)	0.449
Anemia	0.0% (0/27)	9.1% (2/22)	0.196
Malignancy	3.7% (1/27)	4.5% (1/22)	1.000
Autoimmune diseases	0.0% (0/27)	4.5% (1/22)	0.449
Symptoms and signs			
Fever	74.1% (20/27)	100.0% (22/22)	**0.030**
Fatigue	55.6% (15/27)	54.5% (12/22)	0.944
Dyspnea	66.7% (18/27)	54.5% (12/22)	0.386
Cough	74.1% (20/27)	63.6% (14/22)	0.430
Sputum production	55.6% (15/27)	54.5% (12/22)	0.944
Pharyngeal pain	18.5% (5/27)	9.1% (2/22)	0.598
Abdominal pain	29.6% (8/27)	18.2% (4/22)	0.354
Diarrhea	37.0% (10/27)	22.7% (5/22)	0.280
Headache	22.2% (6/27)	18.2% (4/22)	1.000
Dizziness	11.1% (3/27)	4.5% (1/22)	0.756
Nausea	37.0% (10/27)	18.2% (4/22)	0.146
Vomiting	33.3% (9/27)	13.6% (3/22)	0.111
Anorexia	29.6% (8/27)	9.1% (2/22)	0.156
Myalgia	22.2% (6/27)	18.2% (4/22)	1.000
Disease severity status			
Severe/Critically severe	11.1% (3/27)/88.9% (24/27)	36.4% (8/22)/63.6% (14/22)	0.078
Treatment			
MV	80.8% (21/26)	50.0% (11/22)	**0.024**
Invasive MV	100.0% (21/21)	72.7% (8/11)	0.061
PEEP (cm H_2_O)	12.0 (10.0–14.0) (21)	13.0 (12.0–14.0) (8)	0.134
PaCO_2_ (mmHg)	49.0 (44.0–57.3) (18)	48.9 (42.0–64.5) (10)	0.944
Oxygenation index (mmHg)	128.3 (87.1–254.5) (17)	136.4 (62.3–303.5) (6)	0.865
Lactate (mmol/L)	2.1 (1.6–3.1) (10)	2.5 (0.5–3.6) (6)	0.875
Disease outcome			
ICU discharge	11.1% (3/27)	50.0% (11/22)	**0.003**
Time from diagnosis to ICU discharge (days)	29.0 (NA) (3)	24.0 (17.0–35.0) (11)	0.582
Death	88.9% (24/27)	50.0% (11/22)	**0.003**
Time from diagnosis to death (days)	17.5 (12.0–24.0) (24)	23.0 (16.0–28.0) (11)	0.405

Bold values indicate statistical significance

Data are presented as median (Q1–Q3) (N) or % (n/N). COVID-19: coronavirus disease 2019, ICU, intensive care unit; MV, mechanical ventilation; PEEP, positive end-expiratory pressure; PaCO_2_, partial pressure of carbon dioxide

**Table 4:** Laboratory parameters of COVID-19 patients stratified by Syndecan-1 level at ICU admission (cutoff: 813.8 ng/mL)

	Syndecan-1		P
	High level (N = 27)	Low level (N = 22)	
Tissue and endothelial damage			
Thrombomodulin (ng/mL)	5.1 (3.9–6.0) (27)	3.8 (3.3–4.2) (22)	**0.002**
Inflammation			
IL-1β (pg/mL) ↑	9.1% (1/11)	25.0% (2/8)	0.763
IL-2R (U/mL)	1284.0 (469.0–2029.0) (11)	1052.0 (639.5–1262.0) (9)	0.309
IL-6 (pg/mL)	133.1 (52.6–343.1) (13)	18.0 (8.7–97.9) (12)	**0.003**
IL-8 (pg/mL)	79.3 (11.5–177.0) (11)	24.0 (13.9–57.6) (8)	0.238
IL-10 (pg/mL)	17.0 (2.5–33.7) (11)	4.3 (2.5–13.3) (8)	0.075
TNFα (pg/mL)	16.9 (5.0–33.8) (10)	7.5 (6.3–9.5) (7)	**0.042**
Hematologic			
White blood cell count (× 10^9^/L)	10.3 (6.6–19.0) (27)	11.4 (7.8–16.1) (19)	0.973
Neutrophil count (× 10^9^/L)	9.8 (5.9–18.5) (26)	8.7 (6.2–15.0) (19)	0.573
Lymphocyte count (× 10^9^/L)	0.5 (0.3–0.7) (27)	0.6 (0.4–1.0) (20)	0.079
Red blood cell count (× 10^12^/L) ↓	61.5% (16/26)	40.0% (8/20)	0.147
Hemoglobin (g/L) ↓	81.5% (22/27)	75.0% (15/20)	0.860
Hematocrit (%) ↓	61.5% (16/26)	35.0% (7/20)	0.074
Platelet count (× 10^9^/L)	73.0 (40.0–141.0) (27)	171.0 (111.0–247.0) (19)	**0.002**
Biochemical			
Glucose (mmol/L)	10.5 (7.6–13.8) (26)	9.3 (7.1–12.5) (19)	0.662
Total cholesterol (mmol/L)	2.7 (2.3–3.4) (26)	3.3 (2.8–3.8) (20)	**0.031**
HsCRP (mg/L)	113.9 (66.5–193.0) (20)	51.7 (17.9–94.2) (15)	**0.043**
High-sensitive cardiac troponin I (pg/mL) ↑	69.2% (18/26)	68.4% (13/19)	0.954
Myoglobin (ng/mL) ↑	69.2% (18/26)	21.1% (4/19)	**0.001**
AST (U/L) ↑	50.0% (13/26)	26.3% (5/19)	0.109
LDH (U/L) ↑	96.3% (26/27)	90.0% (18/20)	0.787
CK (U/L) ↑	50.0% (7/14)	14.3% (1/7)	0.266
CK-MB (ng/mL) ↑	50.0% (13/26)	0.0% (0/19)	** < 0.001**
NT-proBNP (pg/mL) ↑	88.0% (22/25)	78.9% (15/19)	0.691
Ferritin (μg/L) ↑	100.0% (5/5)	83.3% (5/6)	1.000
Coagulation			
Prothrombin time (s)	17.3 (15.8–21.3) (27)	15.1 (13.9–15.9) (18)	** < 0.001**
Prothrombin activity (%)	58.0 (41.8–70.0) (26)	76.0 (69.5–91.3) (18)	** < 0.001**
INR	1.4 (1.3–1.8) (26)	1.2 (1.1–1.3) (18)	** < 0.001**
Fibrinogen (g/L)	3.6 (2.8–5.1) (27)	4.5 (3.5–5.4) (18)	0.397
APTT (s)	45.4 (39.3–57.4) (27)	40.3 (33.9–44.0) (18)	**0.011**
Thrombin time (s)	15.5 (14.7–17.7) (27)	15.4 (14.7–16.0) (18)	0.437
D-dimer (μg/mL FEU)	7.1 (2.5–21.0) (26)	9.6 (1.3–18.5) (18)	0.589
Fibrinogen degradation products (μg/mL)	44.7 (14.5–150.0) (12)	55.1 (4.0–77.7) (7)	0.592
Antithrombin (%)	77.0 (57.3–86.5) (14)	82.0 (79.0–93.0) (9)	0.058
Other			
Procalcitonin (ng/mL)	1.8 (0.3–3.5) (10)	0.1 (0.1–0.4) (12)	**0.006**
ESR (mm/h) ↑	75.0% (3/4)	100.0% (5/5)	0.444
aPLs positive	50.0% (2/4)	50.0% (1/2)	1.000

Bold values indicate statistical significance

Data are presented as median (Q1–Q3) (N) or % (n/N)

COVID-19, coronavirus disease 2019; ICU, intensive care unit; IL, interleukin; TNF-α, tumor necrosis factor-alpha; hsCRP, high-sensitivity C-reactive protein; AST, aspartate transaminase; LDH, lactate dehydrogenase; CK, creatine kinase; CK-MB, creatine kinase-myoglobin band; NT-proBNP, N-terminal pro-brain natriuretic peptide; INR, international normalized ratio; APTT, activated partial thromboplastin time; ESR, erythrocyte sedimentation rate; aPLs, antiphospholipid antibodies

### Associations between Syndecan-1 levels and laboratory parameters

**Fig. 3:**
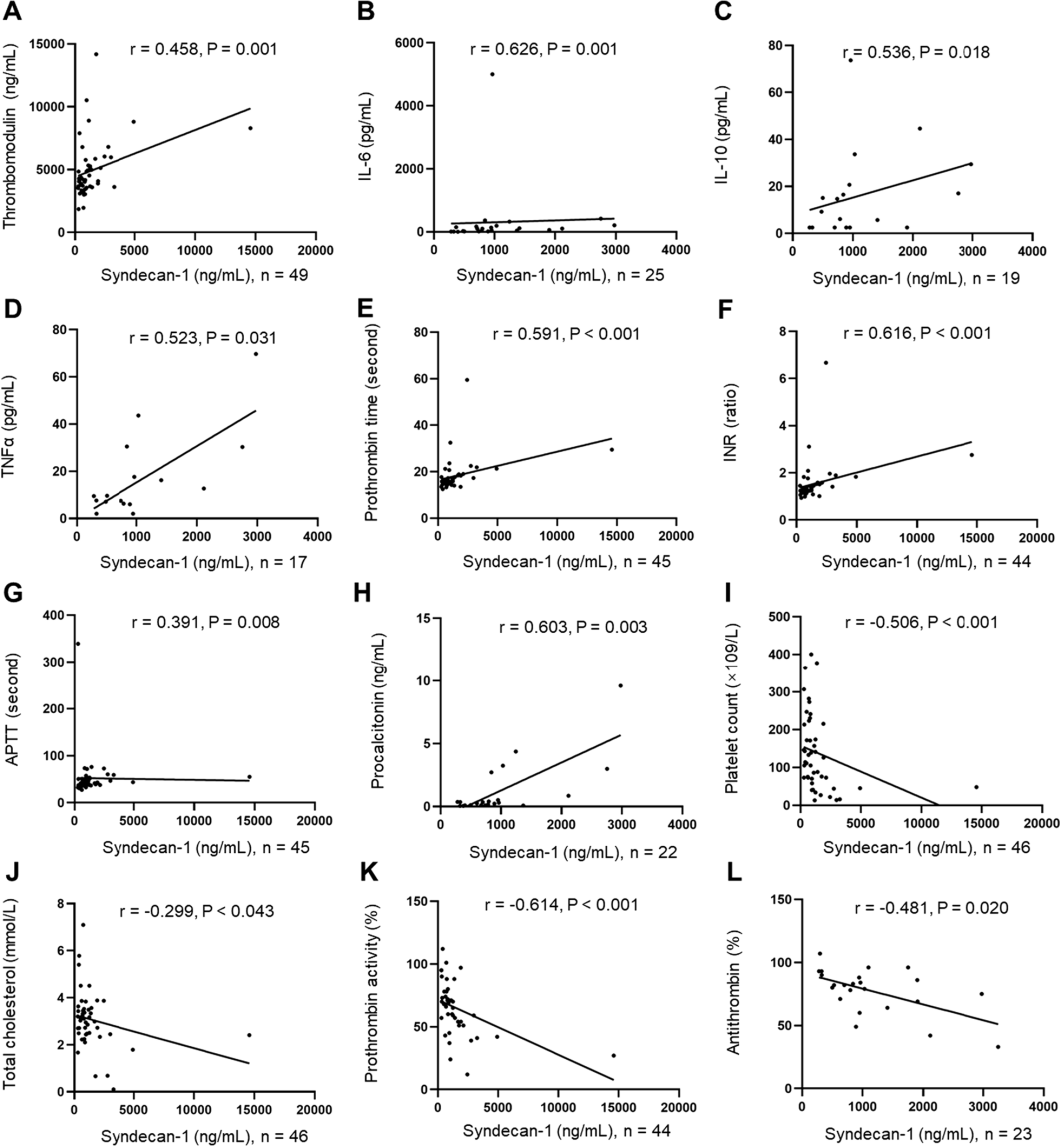
Associations between Syndecan-1 levels and laboratory parameters. **A**–**G** The level of Syndecan-1 was significantly and positively associated with these laboratory parameters. **I**–**L** The level of Syndecan-1 was significantly and negatively associated with these laboratory parameters

The association between Syndecan-1 levels and laboratory parameters was analyzed (Additional file 1: Table S2). The level of Syndecan-1 was significantly and positively associated with thrombomodulin, IL-6, IL-10, TNFα, prothrombin time, INR, APTT and procalcitonin, and negatively associated with platelet count, total cholesterol, prothrombin activity and antithrombin (Fig. 3).

### Dynamics of Syndecan-1 levels in COVID-19 patients

**Fig. 4:**
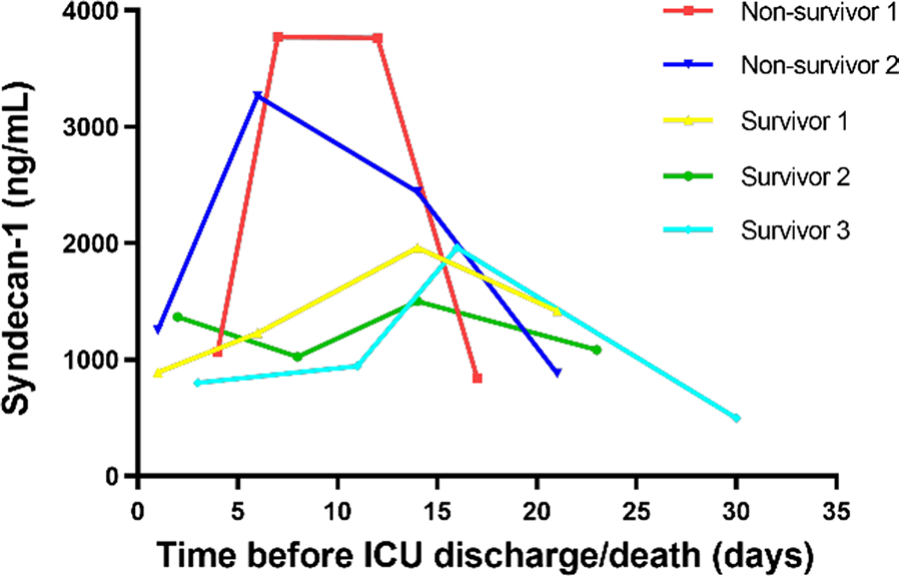
Dynamics of the levels of syndecan-1 in five patients with COVID-19

We had data on 5 patients (2 non-survivors and 3 survivors) on the dynamics of Syndecan-1 during patients stayed in the ICU (Fig. 4). Interestingly, the 2 non-survivors had very large changes over time, with a threefold to fourfold increase within 10 days of admission, followed by declines. The 3 survivors had relatively minor changes over time.

## Discussion

In this study, we measured two endothelial damage biomarkers (Syndecan-1 and thrombomodulin) in sera obtained from COVID-19 patients who were admitted to an ICU in Wuhan, China. Patients were enrolled in the early stage of COVID-19 outbreak, when underdiagnosis or undertreatment of this disorder may explain the high incidence of mortalities. Our data indicate a high level of Syndecan-1 is associated with increased mortality, and is associated with increased levels of thrombomodulin, pro-inflammatory cytokines, hsCRP, and procalcitonin, which suggests the presence of serious endothelial damage, inflammation, and sepsis in these patients. In addition, although our data are limited, non-survivors had significant increases in Syndecan-1 levels over time. Overall, our results suggest that Syndecan-1 could be used as a biomarker for monitoring COVID-19 progression, and possibly that prevention of glycocalyx destruction could be a new method for treatment of COVID-19.

An elevated serum level of Syndecan-1 is usually considered a consequence of the loss of endothelial glycocalyx (Johansson et al. 2011). The endothelial glycocalyx is located on the luminal side of blood vessels, and is mainly comprised of proteoglycans, glycosaminoglycans, and glycoproteins. This glycocalyx layer prevents direct contact of blood cells and endothelial vascular cells, and inflammation can induce endothelial glycocalyx degradation (Uchimido et al. 2019). A loss of the integrity of the endothelial glycocalyx disrupts homeostasis of the vasculature, leading to increased vascular permeability, unregulated vasodilation, microvessel thrombosis, and exposure of endothelial cells to circulating blood cells, all of which culminate in endothelial damage, inflammation, and coagulopathy (Uchimido et al. 2019; Ostrowski and Johansson 2012; Haywood-Watson 2011). The exact mechanism responsible for the increased serum level of Syndecan-1 during the progression of COVID-19 is poor understood. A study reported hypoxia or deletion of syndecan-1 results in reduced binding of the receptor-binding domain of SARS-CoV-2 to epithelial cells (Prieto-Fernandez et al. 2021).

Our study found that non-survivors had high levels of Syndecan-1. There was also a trend toward higher thrombomodulin in non-survivors, but the difference did not achieve statistical significance likely because of the small sample size and insufficient power to detect such a difference. We also found patients with high Syndecan-1 levels had high levels of thrombomodulin, which confirms the presence of endothelial damage in these patients. In agreement, recent studies showed that COVID-19 infection was associated with endothelial damage (Karampoor 2021; Suzuki et al. 2021; Fraser 2020a; Goshua et al. 2020; Escher et al. 2020; Mobayen 2021; Kim et al. 2021). Additionally, we found that the levels of IL-6, TNFα, hsCRP, and procalcitonin were higher in patients with high levels of Syndecan-1, implying that this group of patients have more proinflammatory cytokines and more severe inflammation. Patients with high Syndecan-1 levels also had decreased platelet counts, possibly the result of thrombus formation. There is evidence that the presence of a cytokine storm in COVID-19 patients increases the risk for disease severity and mortality (Bassetti et al. 2020; Huang et al. 2020; Fraser 2020b). Hypercoagulation is another distinctive feature of patients with severe and critical COVID-19 and, increased inflammatory status and endothelial dysfunction are major inducers of hypercoagulation (Cao and Li 2020). Thus, we hypothesized that there may be association among Syndecan-1, proinflammatory cytokines, inflammation, endothelial damage and hypercoagulation in COVID-19 patients, which requires further investigation.

A higher serum level of Syndecan-1 indicates more severe degradation of the endothelial glycocalyx and increased endothelial injury. The greater mortality in patients with high levels of this marker suggests that preservation of glycocalyx function may have therapeutic efficacy in treatment of COVID-19. Several recent studies have examined the effects of protection and re-synthesis of the glycocalyx on inflammatory diseases, but there are not yet any clear conclusions. For example, one study showed that hydrocortisone and antithrombin prevented the endothelial glycocalyx from inflammatory degradation that was initiated by administration of TNFα to guinea pig hearts (Chappell et al. 2009). Another study of a mouse model of sepsis showed that sulodexide accelerated regeneration of the endothelial glycocalyx by reducing vascular permeability (Song et al. 2017). A clinical study of patients with type 2 diabetes mellitus found that oral sulodexide administration improved glycocalyx structure and function in the sublingual and retinal microvasculature (Broekhuizen et al. 2010). A study of a canine model of septic shock found that unfractionated heparin prevented shedding of the glycocalyx by reducing inflammation (Yini et al. 2015). A study of a mouse model of hemorrhagic shock showed that administration of fresh frozen plasma restored pulmonary Syndecan-1 expression, and also inhibited inflammation and endothelial cell hyperpermeability (Peng et al. 2013). Although these studies suggest that prevention or reversal of endothelial glycocalyx damage has therapeutic potential, the efficacy of these interventions in clinical settings remain unknown.

This study has several limitations. First, the sample size of this retrospective study was too small for multivariate analysis. Second, prospective studies with large sample sizes are needed to validate our findings. Finally, the dynamics of serum Syndecan-1 level were studied only in 5 patients. Studies with more patients are required to examine the association between Syndecan-1 and the disease state of COVID-19, and the relationship between Syndecan-1 and the mechanism that leads to severe conditions of COVID-19 is required as well.

## Conclusions

We found that patients with more severe COVID-19 developed endothelial damage, inflammation, and coagulation abnormalities. A high serum level of Syndecan-1 was associated with increased mortality in patients admitted to an ICU with COVID-19.

## Supplementary Information

**Additional file 1:** **Table S1.** Normal reference ranges of parameters included in this study. **Table S2 **Bivariate associations between Syndecan-1 and laboratory parameters.

## Data Availability

All data generated or analyzed during this study are included in this published article.

## Abbreviations

COVID-19: Coronavirus disease 2019
ICU: Intensive care unit
SARS-CoV-2: Severe acute respiratory syndrome coronavirus 2
ACE2: Angiotensin converting enzyme 2
Ang: Angiotensin
RT-PCR: Reverse-transcriptase polymerase-chain-reaction
ELISA: Enzyme-linked immunosorbent assay
ROC: Receiver operating characteristic
IL: Interleukin
HsCRP: High sensitivity C-reactive protein
INR: International normalized ratio
AUC: Area under curve
TNF: Tumor necrosis factor
CK-MB: Creatinine kinase myocardial band
APTT: Activated partial thromboplastin time
