# Supplementary material for: Syndecan-1, an indicator of endothelial glycocalyx degradation, predicts outcome of patients admitted to an ICU with COVID-19
Source: Mol Med. 2021 Dec 3;27:151. doi: 10.1186/s10020-021-00412-1 (PMC8640509; doi:10.1186/s10020-021-00412-1)
Supplement: Supplementary file 1 — Additional file 1: Table S1. Normal reference ranges of parameters included in this study. Table S2 Bivariate associations between Syndecan-1 and laboratory parameters. [file 10020_2021_412_MOESM1_ESM.docx]

**Table S1** Normal reference ranges of parameters included in this study

|  | **Reference range** |
| --- | --- |
| **Invasive MV** |  |
| PaCO_2_ (mmHg) | 35–45 |
| Oxygenation index (mmHg) | 400–500 |
| Lactate (mmol/L) | 0.5–1.6 |
| **Inflammation** |  |
| IL-1β (pg/mL) ↑ | <5 |
| IL-2R (U/mL) | 223–710 |
| IL-6 (pg/mL) | <7 |
| IL-8 (pg/mL) | <62 |
| IL-10 (pg/mL) | <9.1 |
| TNFα (pg/mL) | <8.1 |
| **Hematologic** |  |
| White blood cell count (×10^9^/L) | 3.5–9.5 |
| Neutrophil count (×10^9^/L) | 1.8–6.3 |
| Lymphocyte count (×10^9^/L) | 1.1–3.2 |
| Red blood cell count (×10^12^/L) ↓ | Male 4.3–5.8; Female 3.8–5.1 |
| Hemoglobin (g/L) ↓ | Male 130–175; Female 115–150 |
| Hematocrit (%) ↓ | Male 40–50; Female 35–45 |
| Platelet count (×10^9^/L) | 125–350 |
| **Biochemical** |  |
| Glucose (mmol/L) | 4.11–6.05 |
| Total cholesterol (mmol/L) | <5.18 |
| HsCRP (mg/L) | <1 |
| High-sensitive cardiac troponin I (pg/mL) ↑ | Male ≤34.2; Female ≤15.6 |
| Myoglobin (ng/mL) ↑ | Male ≤154.9; Female ≤106 |
| AST (U/L) ↑ | Male ≤40; Female ≤32 |
| LDH (U/L) ↑ | Male 135–225; Female 135–214 |
| CK (U/L) ↑ | Male ≤190; Female ≤170 |
| CK-MB (ng/mL) ↑ | Male ≤7.2; Female ≤3.4 |
| NT-proBNP (pg/mL) ↑ | Male <241; Female <285 |
| Ferritin (μg/L) ↑ | Male 30–400; Female 15–150 |
| **Coagulation** |  |
| Prothrombin time (s) | 11.5–14.5 |
| Prothrombin activity (%) | 75-125 |
| INR | 0.8–1.2 |
| Fibrinogen (g/L) | 2–4 |
| APTT (s) | 29–42 |
| Thrombin time (s) | 14–19 |
| D-dimer (μg/mL FEU) | <0.5 |
| Fibrinogen degradation products (μg/mL) | <5 |
| Antithrombin (%) | 80–120 |
| **Other** |  |
| Procalcitonin (ng/mL) | <0.05 |
| ESR (mm/h) ↑ | Male 0–15; Female 0–20 |
| aPLs positive | Negative |

MV: mechanical ventilation, PaCO2: partial pressure of carbon dioxide, IL: interleukin, TNF-α: tumor necrosis factor-alpha, hsCRP: high-sensitivity C-reactive protein, AST: aspartate transaminase, LDH: lactate dehydrogenase, CK: creatine kinase, CK-MB: creatine kinase-myoglobin band, NT-proBNP: N-terminal pro-brain natriuretic peptide, INR: international normalized ratio, APTT: activated partial thromboplastin time, ESR:  erythrocyte sedimentation rate, aPLs: antiphospholipid antibodies.

**Table S2** Bivariate associations between Syndecan-1 and laboratory parameters

|  | **Total (N)** | **r** | **P** |
| --- | --- | --- | --- |
| Thrombomodulin (ng/mL) | 49 | 0.458 | **0.001** |
| IL-2R (U/mL) | 20 | 0.205 | 0.387 |
| IL-6 (pg/mL) | 25 | 0.626 | **0.001** |
| IL-8 (pg/mL) | 19 | 0.432 | 0.065 |
| IL-10 (pg/mL) | 19 | 0.536 | **0.018** |
| TNFα (pg/mL) | 17 | 0.523 | **0.031** |
| White blood cell count (×10^9^/L) | 46 | 0.064 | 0.671 |
| Neutrophil count (×10^9^/L) | 45 | 0.132 | 0.388 |
| Lymphocyte count (×10^9^/L) | 47 | -0.287 | 0.050 |
| Platelet count (×10^9^/L) | 46 | -0.506 | **<0.001** |
| Glucose (mmol/L) | 45 | 0.222 | 0.143 |
| Total cholesterol (mmol/L) | 46 | -0.299 | **0.043** |
| HsCRP (mg/L) | 35 | 0.334 | 0.050 |
| Prothrombin time (second) | 45 | 0.591 | **<0.001** |
| Prothrombin activity (%) | 44 | -0.614 | **<0.001** |
| INR (ratio) | 44 | 0.616 | **<0.001** |
| Fibrinogen (g/L) | 45 | -0.148 | 0.332 |
| APTT (second) | 45 | 0.391 | **0.008** |
| Thrombin time (second) | 45 | 0.103 | 0.503 |
| D-dimer (μg/mL FEU) | 44 | 0.148 | 0.339 |
| Fibrinogen degradation products (μg/mL) | 19 | 0.369 | 0.120 |
| Antithrombin (%) | 23 | -0.481 | **0.020** |
| Procalcitonin (ng/mL) | 22 | 0.603 | **0.003** |

IL: interleukin, TNF-α: tumor necrosis factor-alpha, hsCRP: high-sensitivity C-reactive protein, INR: international normalized ratio, APTT: activated partial thromboplastin time.
